# Phylogenetic analysis of dengue virus reveals the high relatedness between imported and local strains during the 2013 dengue outbreak in Yunnan, China: a retrospective analysis

**DOI:** 10.1186/s12879-015-0908-x

**Published:** 2015-03-21

**Authors:** Binghui Wang, Yaping Li, Yue Feng, Hongning Zhou, Yaobo Liang, Jiejie Dai, Weihong Qin, Yunzhang Hu, Yajuan Wang, Li Zhang, Zulqarnain Baloch, Henglin Yang, Xueshan Xia

**Affiliations:** Faculty of Life Science and Technology, Center for Molecular medicine in Yunnan Province, Kunming University of Science and Technology, Kunming, China; Care center for International travel health in Yunnan, Kunming, China; Yunnan Research Institute of parasitic disease control, Kunming, China; Institute of Molecular Biology, Chinese Academy of Medical Sciences & Peking Union Medical College, Kunming, China

**Keywords:** Dengue virus, Viral outbreak, Phylogenetic analysis, Bayesian analysis, Imported infection

## Abstract

**Background:**

An outbreak of dengue virus (DENV) occurred in Yunnan province. More than 2,000 individuals were infected from August to November 2013. In this study, we aimed to characterize the origin and prevalence of DENV in Dehong prefecture of Yunnan province using phylogenetic and evolutionary analyses of DENV strains collected from local patients and foreign travelers.

**Methods:**

A total of 41 DENV-positive serum samples were randomly collected from travelers who entered China at Ruili port or local patients with dengue fever in Dehong prefecture of Yunnan province, China. The envelope (E) gene of DENV was amplified and sequenced. The distributions and evolutionary characteristics of different genotypes were elucidated by phylogenetic and Bayesian analyses.

**Results:**

Phylogenetically, all of the 41 DENV-positive samples could be classified into genotype I (43.9%) of serotype DENV-1 and the Asian I genotype (56.1%) of serotype DENV-2. DENV strains derived from local patients and foreign travelers were scattered equally within these two clusters. Furthermore, the DENV strains from the two populations exhibited high relatedness based on evolutionary characteristics.

**Conclusions:**

These results suggested that imported and local DENV strains occurring during the dengue outbreak in 2013 were highly related. Additionally, these data may suggest that this dengue outbreak was caused by a newly imported infection from the neighboring country of Myanmar.

## Background

Dengue fever is a mosquito-borne disease caused by the dengue virus (DENV). Due to frequent human migration and the vast distribution of mosquito vectors, the distribution of DENV has grown dramatically worldwide in recent years [1]. An estimated 50–100 million cases of dengue fever occur annually in tropical and subtropical areas [1,2]. In China, 20% of the land area is considered to have a tropical climate. Moreover, because of the high population of China, dengue fever represents a substantial health concern in this country.

Serologically, DENV can be classified into five immunologically related but genetically and antigenically distinct serotypes (DENV-1–5) [3,4]. Each serotype may be subclassified into several distinct phylogenetic clusters or genotypes [5]. The various serotypes or genotypes have different epidemic potential in different geographic regions [6]. For example, DENV-1 and DENV-2 are reported to be prevalent throughout the world, while DENV-3 and DENV-4 appear to be limited to East Asian and Southeast Asian countries [7-10]. Additionally, a new serotype of DENV-5 was recently identified in Malaysia [4]. Thus, identification of serotypes may provide insights into the origins of DENV outbreaks.

Dengue epidemics have been reported in Southern China since 1978 [11]. The incidence of DENV infection is increasing in the coastal provinces of China; this outbreak is considered an imported epidemic from neighboring Southeast Asian countries [12-15], which have been recognized as the geographical regions from which the identified serotypes originated [16]. Yunnan province is located in Southwestern China, which borders the countries of Southeast Asia where dengue fever is epidemic. Previously, imported cases of DENV infection were sporadically reported in bordering areas of Yunnan [17,18]. Surprisingly, from August to November 2013, an outbreak of DENV occurred in Dehong and Xishuangbanna prefectures, with more than 2,000 infected individuals. This massive DENV outbreak and the pervasiveness of the mosquito vector (*Aedesalbopictus*) have caused major concerns in the general population. However, the molecular epidemiology of the DENV involved in this outbreak is still unknown.

Therefore, in this study, we aimed to characterize the origin and prevalence of DENV in Dehong prefecture of Yunnan province using phylogenetic and evolutionary analyses of DENV strains collected from local patients and foreign travelers.

## Methods

### Ethics statement

All participants were informed of the aims of the study and procedures involved in study participation at enrolment, and written informed consent was received before sample collection. The study was approved by the Institutional Ethical Committee of Kunming University of Science and Technology.

### Sample collection

During the dengue outbreak in Yunnan province from August to November 2013, a total of 18 imported DENV-positive samples were collected from travelers who entered China at the land port of Ruili. Additionally, 23 samples were randomly collected from patients with dengue fever admitted at the local hospital in Dehong prefecture, the western-most prefecture of Yunnan Province. Detection of DENV NS1 antigen was conducted using a rapid one step dengue fever NS1 test Kit (Blue Cross, Beijing, China) to diagnose DENV infection. Serum samples were separated from the collected blood and stored at –80°C.

### Amplification and sequencing of the envelope (E) gene

RNA was extracted from 100-μL serum samples using a High Pure Viral RNA Kit (Roche, Shanghai, China) according to the manufacturer’s instructions. The entire E gene was amplified by a one-step reverse transcription polymerase chain reaction (RT-PCR) using a One-Step RT-PCR Kit (TaKaRa, Dalian, China) with the following protocol: initial reverse transcription at 50°C for 30 min; 35 cycles of denaturation at 94°C for 30 s, annealing at 50°C for 30 s, and elongation at 72°C for 2.5 min; and a final elongation step at 72°C for 7 min. All serotype-specific primers used in this study were reported previously [19]. PCR products were confirmed by electrophoresis and purified using an Agarose Gel DNA Extraction Kit (TaKaRa). Sequencing was then performed by Invitrogen (Beijing, China). All raw sequences obtained were checked in the Chromas program (http://www.technelysium.com.au). DNA fragments encoding the full-length envelope protein of DENV were submitted to GenBank (accession numbers KJ939367 to KJ939407).

### Phylogenetic analysis

Sequences of the E gene were aligned using the ClustalX program [20] and compared with reference sequences derived from the GenBank database. Serotypes and genotypes were determined by the reconstructed maximum likelihood (ML) tree using MEGA 5.0 software [21] in the Kimura 2 parameter model with gamma distribution and invariant sites. The nodal reliability of the ML trees was assessed by bootstrapping (BS) with 1000 pseudoreplicates.

### Bayesian phylogenetic and phylogeographic analyses

A total of 244 reference sequences of the DENV envelope gene, with sampling dates ranging from 1956 to 2011, were retrieved from GenBank. These sequences represented different geographical regions. Phylogeographic analysis was conducted together with the obtained 41 sequences in the current study. Bayesian coalescent analysis was performed in BEAST v1.6.1 to estimate the mean evolutionary rate of the E gene and the time to the most recent common ancestor (tMRCA) for the most prevalent group using the general time reversible (GTR) + gamma + I substitution model and the relaxed uncorrelated lognormal molecular clock [22]. Convergence of the MCMC sample on the posterior distribution was defined at an effective sample size (ESS) value of greater than 200, which was calculated with Tracer v.1.4 (available at http://beast.bio.ed.ac.uk/Tracer). The maximum clade credibility (MCC) tree was constructed using TreeAnnotator v.1.4.8 and then visualized using FigTree v.1.3.1 (available at http://tree.bio.ed.ac.uk/software/figtree/).

### Statistical analysis

Statistical analysis was conducted using SPSS version 12.0 software (SPSS Inc., Chicago, IL, USA). Characteristics were compared between the groups using χ^2^ tests, and results with *P* values of less than 0.05 were considered statistically significant.

## Results

### Demographic characteristics

During the dengue outbreak from August to November 2013, 246 cases of suspected dengue fever were recorded and treated at local hospitals in Ruili county in Dehong prefecture in Yunnan. Twenty-three samples were randomly collected and determined to be DENV positive. These 23 patients with DENV infection were all local residents according to hospital records. For comparison, 18 DENV-positive serum samples were collected from foreign travelers exhibiting symptoms of dengue fever who entered at Ruili port. Of these 18 travelers from Myanmar, five were Chinese (27.8%) and 13 were Burmese (72.2%). Of the recruited DENV-infected individuals, the median age was 30.3 ± 14.6 years (range, 3–80 years), and 16 persons (39.0%) were female. Twenty (48.8%) patients were engaged in the service industry, 10 (24.4%) were unemployed, five (12.2%) were farmers, four (9.8%) were students, and two (4.9%) were teachers. There were no observed differences in age (*P* > 0.05) or gender (*P* > 0.05) between these two populations.

### Distributions of DENV serotypes and genotypes and phylogenetic relationships between imported and local strains

Phylogenetically, the 41 strains could be classified into two serotypes: 18 DENV-1 strains (43.9%) and 23 DENV-2 strains (56.1%). All 18 of the DENV-1 strains were further subclassified into genotype I, one of the most prevalent genotypes in Southeast Asian countries [23]. Of these, nine strains were from travelers who entered China at Ruili port in Yunnan Province, and the other nine strains were from local patients. Of the 23 DENV-2 strains, nine were from foreign travelers, and 14 were from local patients.

**Figure 1:**
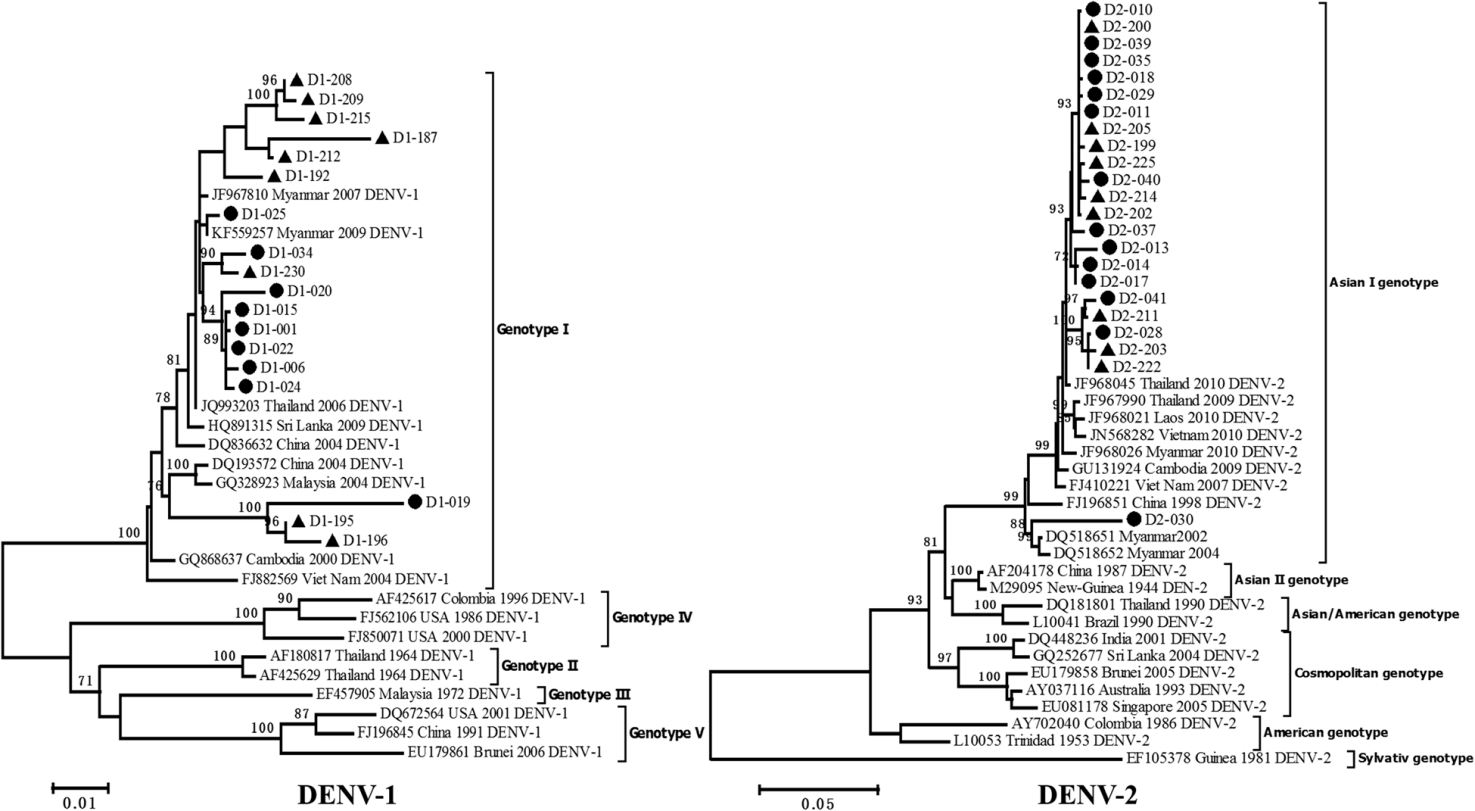
**Phylogenetic tree of DENV-1 and DENV-2.** The phylogenetic tree was constructed by the maximum likelihood method with a Kimura 2 parameter model using MEGA 5.0 software. Bootstrap values were set for 1000 repetitions. The black dots denote strains from the local population, and the black triangles denote strains from foreign travelers. The sequences of reference strains were derived from GenBank (www.ncbi.nlm.nih.gov).

The 18 DENV-1 strains were further divided into two related clusters in the ML tree, each formed randomly by imported or local strains (Figure 1, left). DENV-1 strains from Southeast Asian countries (Thailand, Myanmar, Cambodia, and Vietnam) and southern China were also clustered together with these identified strains, with a BS value of 100. Except for strain D2-030, all of the other imported and local DENV-2 strains in the current study were dispersed equally into one cluster together with the strains from Southeast Asia (Figure 1, right). The genetic distances between our identified DENV strains and strains from Southeast Asian countries observed in the ML tree of DENV-2 were shorter than those between our strains and other Chinese strains (0.015 versus 0.038, respectively). Moreover, for phylogenetic analyses of both DENV-1 and DENV-2, several strains isolated in the current study were clustered together with strains from Myanmar or its neighboring countries. Based on the geographic position of Dehong prefecture, which is a neighbor of Myanmar, it is reasonable to suspect that cross-border transmission contributes to the spread of DENV and may have caused the dengue outbreak in Dehong prefecture, Yunnan in 2013.

### Evolutionary characteristics of the identified DENV strains

Bayesian coalescent analysis was performed to further illustrate the evolutionary characteristics of the DENV circulating in this region. In the chosen GTR + G4 + I and uncorrelated lognormal molecular clock model, the mean values of the evolutionary rates in the DENV E gene were estimated to be 8.3435 × 10^−4^ and 8.1564 × 10^−4^ substitutions/site/year for DENV-1 and DENV-2, respectively.

**Figure 2:**
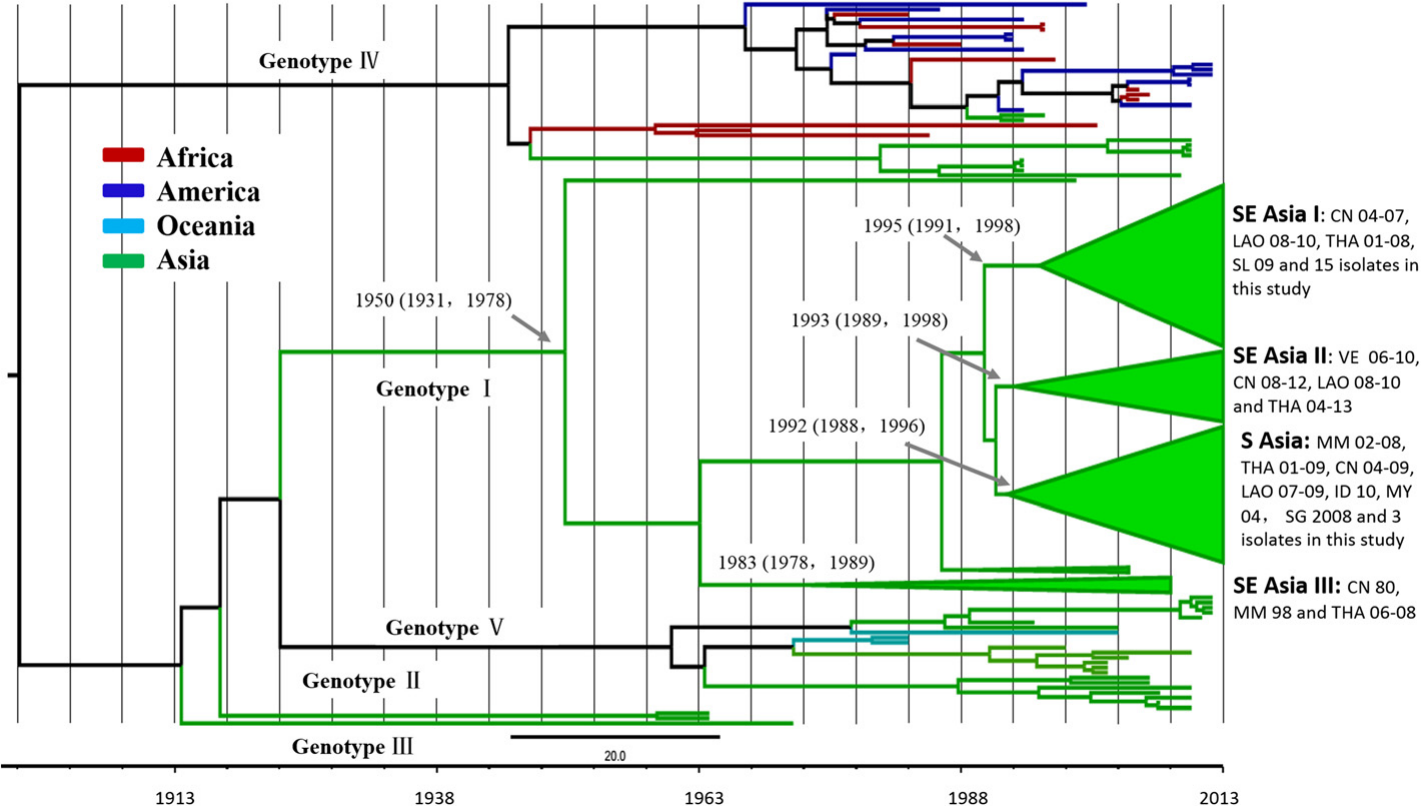
**Maximum clade credibility (MCC) tree for DENV-1 with collapsed branches corresponding to the genotypes/major subclades.** The countries (ISO 3166-1-alpha-2 codes) and sampling times of the stains are also indicated. The numbers correspond to the posterior supports in square brackets and the time to the most recent common ancestor (TMRCA) estimates of key nodes.

A total of 244 E gene sequences of DENV-1 and DENV-2 were used as reference strains for subsequent evolutionary analysis. For DENV-1, most strains of DENV genotypes I, II, III, and V were from Asia, while some strains of DENV genotype IV were from African and American countries (Figure 2). In TMRCA analysis based on the estimated nucleotide substitution value, the DENV-1 genotype I was estimated to be the earliest ancestor, emerging at least 63 years ago (95% HPD, years 1931–1978). Four lineages defined as Southeast Asia I (SEA I), Southeast Asia II (SEA II), Southeast Asia III (SEA III), and South Asia (SA) were differentiated in chronological order from 1983 to 1995. In the current study, 15 strains were clustered into the SEA I lineage together with the stains from China, Laos, Thailand, and Sri Lanka. The other three local DENV strains evolved in the SA lineage together with strains from Myanmar, Thailand, China, Laos, Indonesia, Malaysia, and Singapore.

**Figure 3:**
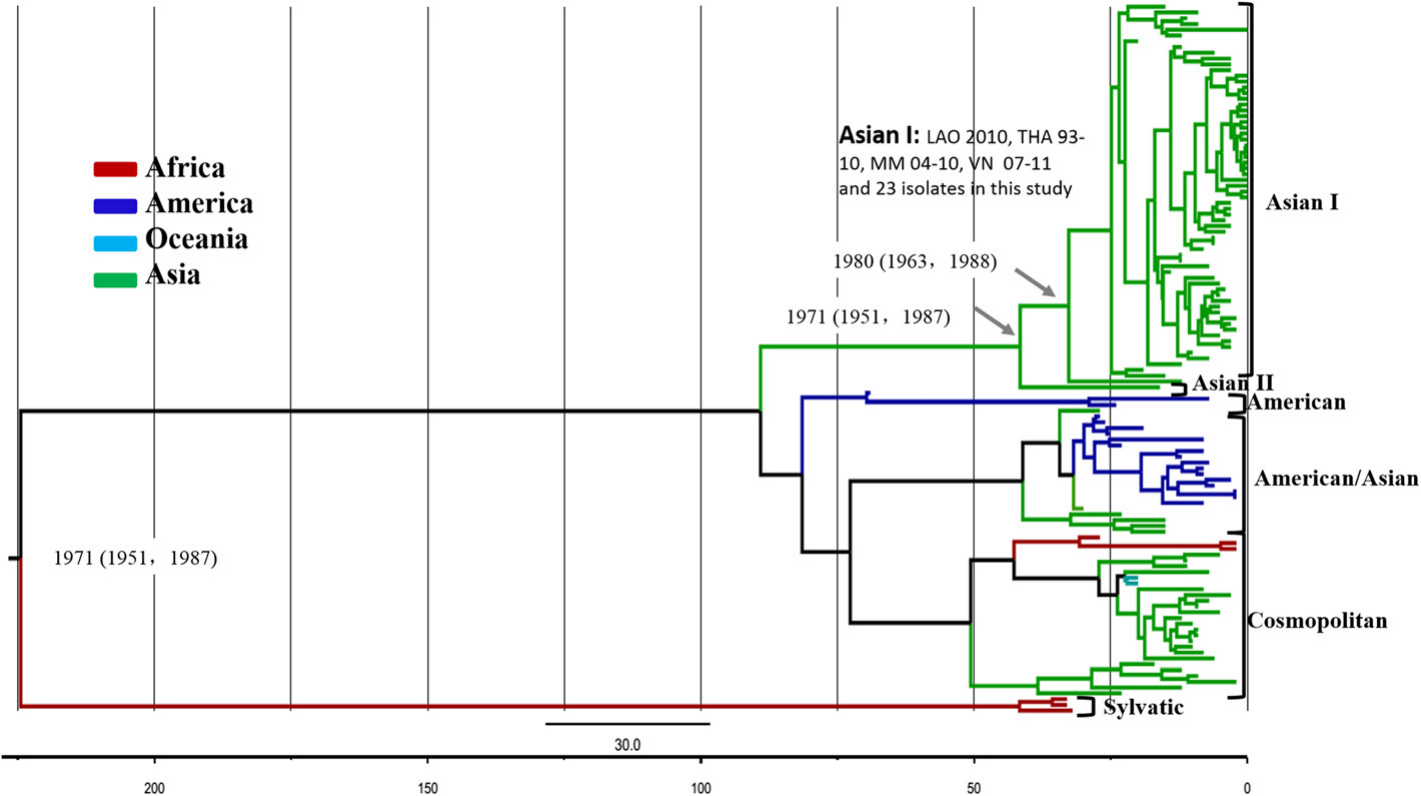
**Maximum clade credibility (MCC) tree for DENV-2 with collapsed branches corresponding to the genotypes/major subclades.** The countries (ISO 3166-1-alpha-2 Codes) and sampling times of the stains were also indicated. The numbers correspond to the posterior supports in square brackets and the time to the most recent common ancestor (TMRCA) estimates of key nodes.

Evolutionary analysis of DENV-2 was performed using the same model. The MCC tree showed that six genotypes could be classified (Figure 3), consistent with previous reports [24]. These Asian strains were found in Cosmopolitan, American/Asian, Asian I, and Asian II genotypes; in contrast, the genotype distributions of strains from other continents were obviously narrower. Asian genotypes seemed to be the most prevalent clade for DENV-2, and no independent sublineage was obvious in this clade. The major strains of the DENV-2 Asian I genotype circulated in Southeast Asian countries, including Laos, Thailand, Myanmar, and Vietnam. Notably, all the DENV-2 strains in the current study were subclassified into the Asian I genotype, which emerged about 33 years ago (95% HPD, years 1963–1988).

## Discussion

Yunnan province continues to face challenges with dengue fever outbreak due to problems with mosquitos and overpopulation, as well as the close proximity to regions with high incidences of DENV infection. Sporadic epidemics of dengue fever have been reported frequently in recent years, leading up to the outbreak of DENV in 2013 in Yunnan province. During this outbreak, more than 2,000 DENV infections were detected from August to November 2013, and more than 1,600 DENV-positive samples were collected from seven prefectures. Among them, Xishuangbannan (the southern-most prefecture of Yunnan Province) and Dehong (the western-most prefecture of Yunnan province) experienced the most infections, with 1320 and 270 cases of DENV infection, respectively. In this study, we assessed the phylogenetic and evolutionary characteristics of the strains involved in this outbreak in order to provide insights into the origins of the outbreak. Our data showed that the infections were likely to have originated from foreign travelers rather than within Yunnan province.

Through simultaneous sampling from travelers and local patients with dengue fever, coupled with the subsequent phylogenetic and evolutionary analyses, we found that there were no significant differences in the distributions of different DENV serotypes and genotypes between these two populations. These findings revealed the high relatedness between imported and local DENV strains, which was further supported by their equal dispersion in the lineage of the ML tree and the observed short genetic distance. Based on our knowledge of the historical imported transmission of DENV in Yunnan, it is reasonable to propose that the dengue outbreak in 2013 was caused by imported infection. Furthermore, both the DENV-1 and DENV-2 strains in the current study formed compact clusters with strains from neighboring Southeast Asian countries, confirming the impact of imported infection on the local dengue outbreak in Yunnan province. Therefore, it is necessary to monitor DENV transmission, particularly in foreign travelers, to prevent the spread of DENV. However, the DENV strains recently identified in Guangdong province have been shown to exhibit higher serological and genetic diversity [23]. This distinct characteristic of this DENV prevalence compared with the outbreak in Yunnan is thought to be related to the different import routes of DENV or by locally circulating serotypes or genotypes.

For evolutionary analysis, we applied a GTR model with a discretized gamma distributed across site rate variation (GTR + Γ4) substitution model and a relaxed (uncorrelated lognormal) molecular clock model [22]. In this model, the estimated E gene evolutionary rates of DENV-1 and DENV-2 were consistent with previous reports [25-27]. TMRCA analysis estimated the origin to have been 63 years ago (i.e., the year 1950) for DENV-1 genotype I, similar to the results of Villabona’s evolutionary analysis of DENV-1 (i.e., originating in the year 1943) [28]. DENV-1 is the predominant genotype thought to have originated from Polynesia, with subsequent spread to and circulation in Indochina and East and Southeast Asian countries [29].

In TMRCA analysis, four relatively independent lineages of genotype I were observed. The SEA I, SEA II, and SA lineages were estimated to have originated around the same period (within the 1990s), later than the SEA III lineage (early 1980s). The genotype I strains in the current study were distributed in the younger lineages of SEA I (15 cases) and SA (thee cases). All of the discovered DENV-2 strains reported in this study were grouped within the Asian genotype II together with strains from Southeast and East Asian countries. The evolutionary characteristics of other genotypes of DENV-2 have been documented [7,30-38]; however, little is known about Asian genotype II. In this study, Bayesian coalescent analysis based on a large number of reference strains, particularly Asian genotype II, showed that the DENV-2 genotype emerged about 33 years ago. No obvious independent lineage of this genotype was subclassified, indicating the relatively limited epidemic of this genotype.

A local outbreak of dengue in southern China was reported to be caused by one Chinese traveler with DENV-2 infection who was returning from India [39,40]. The environment in Yunnan province is suitable for the circulation of DENV, with a tropical to subtropical climate and large populations of mosquitos. Therefore, it is reasonable to assume that the imported cases from neighboring Myanmar caused the dengue outbreak in Yunnan in 2013.

## Conclusions

In summary, the phylogenetic and evolutionary characteristics of the DENV strains during the 2013 dengue outbreak in Yunnan province revealed the close relationship between DENV strains from local patients in Dehong prefecture and strains from foreign travelers who entered at Ruili port from Myanmar. Although our data appear to support that the imported DENV strains from surrounding countries led to the local dengue outbreak, further large-scale studies are required to confirm this hypothesis. The findings in the current study may provide useful epidemic information for dengue prevention and control in China.
